# Self‐Assembled Nanostructured Lipid Systems: Is There a Link between Structure and Cytotoxicity?

**DOI:** 10.1002/advs.201801223

**Published:** 2018-11-12

**Authors:** Angel Tan, Linda Hong, Joanne D. Du, Ben J. Boyd

**Affiliations:** ^1^ ARC Centre of Excellence in Convergent Bio‐Nano Science and Technology Drug Delivery, Disposition and Dynamics Monash Institute of Pharmaceutical Sciences Monash University, Parkville Campus 381 Royal Parade Parkville VIC 3052 Australia

**Keywords:** cell–nanoparticle interactions, drug delivery systems, liquid crystalline lipids, self‐assembled nanostructures, tissue cytotoxicity

## Abstract

Self‐assembly of lipid‐based liquid crystalline (LLC) nanoparticles is a formulation art arising from the hydrophilic–lipophilic qualities and the geometric packing of amphiphilic lipid molecules in an aqueous environment. The diversity of commercialized amphiphilic lipids and an increased understanding of the physicochemical factors dictating their membrane curvature has enabled versatile architectural design and engineering of LLC nanoparticles. While these exotic nanostructured materials are hypothesized to form the next generation of smart therapeutics for a broad field of biomedical applications, biological knowledge particularly on the systemic biocompatibility or cytotoxicity of LLC materials remains unclear. Here, an overview on the interactions between LLCs of different internal nanostructures and biological components (including soluble plasma constituents, blood cells, and isolated tissue cell lines) is provided. Factors affecting cell–nanoparticle tolerability such as the type of lipids, type of steric stabilizers, nanoparticle surface charges, and internal nanostructures (or lipid phase behaviors) are elucidated. The mechanisms of cellular uptake and lipid transfer between neighboring membrane domains are also reviewed. A critical analysis of these studies sheds light on future strategies to transform LLC materials into a viable therapeutic entity ideal for internal applications.

## 1 Introduction

### 1.1 Classification and Characterization of Lipid‐Based Liquid Crystalline (LLC) Systems

**Figure 1:**
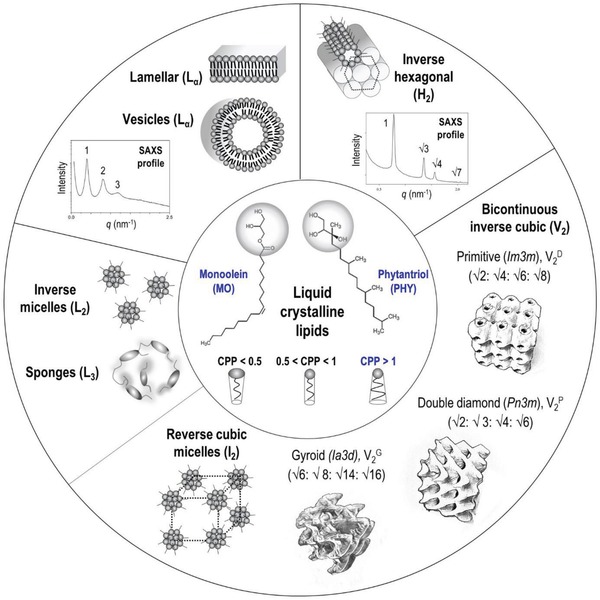
Commonly encountered mesophase structures based on the self‐assembly of liquid crystalline lipids with varying critical packing parameter (CPP), where their characteristic diffraction spacing ratios are typically acquired using small‐angle X‐ray scattering (SAXS) technique.^3^, ^4^, ^5^, ^6^ Drawing not to scale.

Lipid‐based liquid crystalline self‐assemblies are gaining substantial interest in the field of drug delivery due to their ability to incorporate compounds with varying physicochemical properties,^1^ and their complex internal nanoarchitecture that provides a mechanism for sustained release.^2^ Many amphiphilic lipids spontaneously self‐assemble and form thermodynamically stable mesophases on exposure to excess water. Some of the commonly encountered LLC mesophases are: (i) the bilayer lamellar (L_α_) phases; (ii) the 2D inverse hexagonal (H_2_) structure; (iii) the 3D ordered micellar cubic (I_2_ or *Fd3m*) and bicontinuous cubic (V_2_) phases; and (iv) the disordered phases of reverse isotropic micelles (L_2_) and swollen sponges (L_3_) (**Figure** **1**).^3^ Liposomes, spherical bilayer structures resulting from the dispersion of lamellar phase (L_α_), are the simplest case of lyotropic lipid self‐assembly. Cubosomes and hexosomes are dispersed particles of the highly ordered, nonlamellar structures of V_2_ and H_2_ phase, respectively. The type of LLC structure formed is governed by the molecular packing of the lipid molecules, which is reflected by their critical packing parameter (CPP):

(1) $$\mathrm{CPP}=\frac{V_{s}}{a_{0}l}$$

where *V*_s_ is the hydrophobic chain volume, *a*_0_ is the head group area, and *l* is the hydrophobic chain length.^7^ The CPP can be influenced by elemental factors including the concentration and spontaneous curvature of the lipid molecule as well as environmental parameters including temperature,^8^ water content,^9^ ionic strength,^10^ and pH.^11^ A CPP value of <0.5 (i.e., space dominated by the large hydrophilic head groups) generally favors the formation of positively curved spherical micelles; when CPP = 0.5–1, positively curved L_α_ phases (bilayer vesicles) are typically observed, whereas for CPP > 1 (i.e., large hydrophobic tail groups), highly negatively curved inverse phases (such as V_2_, H_2_, and inverse micelles) are formed. Among the many nonlamellar mesophases, cubosomes have been the most studied systems in the biomedical field, with potential applications in MRI imaging, biosensing, therapeutic delivery, and protein crystallization.^12^, ^13^, ^14^, ^15^ The formation of the V_2_ phase requires a delicate balance between the headgroup and tail volume, and this has been typically achieved using monoolein (MO) and phytantriol (PHY) which possess a relatively small nonpolar headgroup and a kink in the hydrophobic tail that promotes formation of the nonlamellar structure. With a CPP > 1, at equilibrium in excess water both of these amphiphiles exhibit the V_2_ phase at ambient temperature, and transform to the H_2_ phase and inverse micelles (L_2_) at higher temperature. The nanostructure and phase transition temperature are programmable via the inclusion of additives into the lipid or aqueous domains [e.g., tetradecane (hydrophobic), polyethylene glycol (PEG, amphiphilic), and sucrose (water‐soluble)]. Extensive reviews on the LLC material selection and design are provided elsewhere.^3^, ^16^

An understanding of LLC phase behavior begins with the characterization of the nanostructure of the phases. A range of structural characterization techniques have been previously reviewed.^4^, ^16^ Cross‐polarized light microscopy (CPLM) is a standard, convenient tool to identify anisotropic bulk materials (e.g., H_2_) by their birefringence appearance, as opposed to the dark view as presented by isotropic phases (e.g., V_2_). For dispersed nanoparticles, cryogenic transmission electron microscopy (cryo‐TEM) enables direct visualization of their morphology and internal nanostructure at relatively low material concentrations.^17^ Diffraction techniques provide a high‐resolution mesoscale approach to qualitatively identify the internal nanostructure and quantitatively determine the dimensions of these mesophases. Small‐angle X‐ray scattering (SAXS) is commonly utilized to obtain 2D SAXS scattering fingerprints of LLC structure. The 2D data are generally integrated into a plot of intensity (*I*) versus scattering vector (*q*), which enables identification of the phase structures by correlating the relative positions and spacing ratios of the signature (“Bragg”) peaks with the Miller indices for each distinct phase.^18^ SAXS also enables the derivation of additional structural information including the interplanar spacing (*d*) between the adjacent diffraction planes, enabling calculation of the mean lattice parameter (*a*) of a system and the dimensions of the water channels; details of these calculations are explained elsewhere.^16^, ^18^ Most importantly, SAXS powered by synchrotron radiation is capable of resolving the dynamic changes in self‐assembled nanostructure within relatively short time frames, and increased access to synchrotron sources has driven much of the recent research in this field.

### 1.2 Engineering of LLC Formulations: Bulk and Nanoparticles

The smart design of LLC formulations is guided by the mode of drug delivery required to address the therapeutic problem at hand: in the case where the problem requires application as a local (sustained‐release or triggered‐release) depot, the self‐assembled material would be prepared in the form of a viscous bulk material for administration as a gel or as an injectable depot. Alternatively, where a fluid system is required, or particles of a sufficiently small size are required (e.g., intravenous injection), the system is amenable to preparation as dispersed nanoparticles in excess aqueous injectable fluids.^5^, ^19^ The bulk “depot” LLC formulations are merely formed via hydration of the lyotropic lipid building blocks, or by addition of a hydrotropic solvent that diffuses away from the depot after administration to allow formation of the bulk phase in situ.

For nanoparticulate formulations, the most common method of engineering is via the top‐down approach, where the precursor bulk lipids are fragmented, dispersed, and stabilized in the presence of an interfacial polymer (e.g., the Pluronic series of block copolymers) in excess water.^20^ The dispersed particles typically exhibit similar internal nanostructures to their bulk counterparts, however, the temperature‐dependent phase transitions may subject to slight variations.^21^ Conventionally, LLC nanoparticles are fabricated through high‐energy processes, notably pulsed ultrasonication,^20^ and high‐shear homogenization.^22^ Despite its simplicity, the high temperatures generated during the processing may risk the integrity of heat‐sensitive components. For liposomes, excessive energy input associated with this method generates multilamellar vesicles, which necessitates additional processing such as extrusion to obtain unilamellar liposomes.^23^

In an effort to decrease the likelihood of heat damage to samples, Spicer et al. developed the hydrotrope‐dilution method.^24^ It involves an initial step where a compatible hydrotrope dissolves the lyotropic lipids in the aqueous solvent to form an isotropic solution. Then, the solubility of the lipid decreases when more aqueous solution is added, such that the lipid precipitates and self‐assembles into the ordered structure via homogeneous nucleation. A key advantage is that particles produced spontaneously upon dilution, and the authors reported that the cubosomes formed are relatively smaller and more stable than the energy‐intensive methods. However, the hydrotrope remaining in the formulation may present impurity issues and require removal by techniques such as dialysis prior to use in further applications.

Another method that does not require the input of high energy is the solvent evaporation approach.^25^ The lipid phase is combined with a volatile solvent (e.g., cyclohexane or limonene) and a surfactant solution (e.g., Pluronic F127) prior to sonicator‐assisted emulsification. Compressed air is bubbled through the mixture to allow complete evaporation of the solvent as confirmed using nuclear magnetic resonance. Furthermore, particle size can also be manipulated with the amount of volatile solvent incorporated. In addition, the phase inversion temperature (PIT) emulsification method may also represent an alternative low‐energy approach for the synthesis of LLC nanoparticles. Originally introduced by Shinoda and Saito in the 1960s, this method takes advantage of the temperature‐induced dehydration of the polar head groups and thus, changes in the interfacial curvature of the amphiphiles.^26^, ^27^, ^28^ Heating near the PIT point of an LLC mixture (e.g., ≥75 °C^28^) drives the formation of negatively curved structures, and a subsequent rapid cooling step (e.g., to 25 °C within 1 min^28^) produces stable LLC nanoparticles.

The aforementioned techniques are bulk‐processing methods, which work with relatively large quantities of materials. Recently, the integration of some of these techniques with microfluidic devices has yielded more uniform nanostructured LLC particles with minimal ingredient consumption.^29^ Coupling of such a microfluidic fabrication platform with synchrotron‐based SAXS is particularly attractive to follow the dynamic mesophase formation and transition within fractions of seconds. Unlike random mixing often associated with bulk processes, microfluidic devices enable precisely controlled mixing of the precursor lipids with the aqueous phase along the microchannels. Self‐assembly of particles occurs via diffusion of materials across one stream to another. Promisingly, Kastner et al. were able to form and tune the size of liposomes by implementing the hydrotrope‐dilution method using a commercially available microfluidic chip.^30^ Alternatively, custom‐designed devices featuring cross‐flows of channels (hydrodynamic flow focusing) can also achieve fine and uniform liposome formation.^31^

Further transformation of these lipid‐based colloids into solid dosage forms may be seen as a viable means to confer physical stability and minimize microbial challenges, as well as to facilitate product transfer and handling.^32^, ^33^ Interestingly, Spicer et al. demonstrated the feasibility of spray drying in converting MO‐based cubosomes into redispersible dry powder precursors with the aid of an encapsulation ingredient, such as starch and dextran.^33^ Instead of using traditional Pluronics, the starch and dextran molecules carry a dual function as the dispersion stabilizer and solid carrier. Rehydration of the powdery agglomerates produced monodisperse particles with an average diameter of 0.6 µm upon gentle stirring. The feasibility of formulating LLC system as a bulk, dispersion or a dry powder will enrich the diversity of dosage form designs for these materials.

## 2 Interactions of LLCs with Biological Components

### 2.1 Artificial Cell Growth Fluids and Blood Serum/Plasma

The majority of studies investigating the structure of nonlamellar LLCs were performed on the as‐prepared formulation using water or phosphate buffered saline (PBS) as the dispersant. When intended for application as an injectable particle formulation, it is imperative to evaluate the phase behaviors and transitions of LLCs in physiologically relevant fluids, including blood plasma, serum, and artificial cell growth media. These media generally contain more complex ionic and protein constituents that may affect the lipid packing parameters, including amino acids (e.g., l‐glutamine), vitamins (soluble B group and fat‐soluble A, D, E, K vitamins), inorganic salts (sodium, potassium, and calcium ions), glucose, and other serum contents including binding proteins (such as albumin), growth factors, hormones, and attachment factors (such as fibronectin).^34^ The fact that LLC matrices have been studied as carriers for proteins, peptides, and nucleic acids signifies their relatively high extent of affinity toward biological molecules.^35^ This section collates the current understanding on how the LLC structural behaviors are affected by the presence of endogenous soluble moieties typically present in cell culture and blood media, which in turn may alter their interactions with blood or tissue cells.

#### 2.1.1 Sugars

Low molecular weight hydrophilic substances, as simple as sugars, can significantly disturb the hydration efficiency of the glycerol head groups of liquid crystalline lipids (or the area of lipid–water contact).^36^, ^37^ In the case of MO (at 30% water content, 20 °C), the addition of sucrose from 1 to 30% (w/v) was found to induce a phase transition toward more negative curvature in the order of cubic *Ia3d* (<2% sucrose) → cubic *Pn3m* (≥5% sucrose) → H_2_ phases (≥10% sucrose).^38^ Both monomeric (i.e., glucose) and polymeric sugars (e.g., sucrose, trehalose, maltose), by virtue of their kosmotropic property, tend to create a competitive microenvironment with the lipid head groups for the hydration water. This results in an augmented bending of the lipid interfacial film toward water, which leads to a decreased lattice parameter (i.e., structure shrinkage) and/or reduced phase transition temperature from the V_2_ to H_2_ phase.^38^, ^39^, ^40^, ^41^, ^42^ When translating to the physiological context, the dilution of lipid:sugar in the blood media needs to be carefully considered according to the physiological levels of glucose ranging from 4.0 × 10^−3^ m (normal fasting state) to 8.0 × 10^−3^ m (normal fed state), or higher in the diseased states (such as diabetes). It is realized that most in vitro investigations have adopted sugar levels of more than 100 times higher than the realistic physiological value (e.g., >100 × 10^−3^ m),^38^, ^39^, ^40^, ^41^, ^42^ therefore the water displacement efficiency and mesophase behavior in the bloodstream may be different from that predicted.

#### 2.1.2 Amino Acids

The presence of naturally occurring amino acids, depending on the polarity of their side chain, can have an impact on the water channels of an ordered LLC nanostructure and in some specific cases, can induce a phase transition.^3^ Chemelli et al. systematically evaluated the interaction of MO‐based nanostructures (at 50% w/v lipids) with five types of amino acids: (polar, neutral) glutamine (146 g mol^−1^); (less polar, aliphatic) alanine (89 g mol^−1^), glycine (75 g mol^−1^); and (nonpolar, aromatic) tryptophan (204 g mol^−1^) and phenylalanine (165 g mol^−1^).^43^ The polar and less polar amino acids were found to act similarly to sugar molecules (as above‐mentioned) in reducing the effective solvation of the lipid glycerol head group, thereby causing a slight shrinkage of the water channels of the original V_2_ structure (i.e., 1–1.7% decrease in the lattice parameter at 2% w/v amino acids). On the contrary, the presence of nonpolar amino acids (at 2–3% w/v) induced significant enlargement in a series of modified MO‐based mesophases, namely, V_2_ (MO), H_2_ (MO/tetradecane 15%), I_2_ (MO/tetradecane 30%), and L_2_ (MO/tetradecane 50%). The enlargement in lattice spacings, that is, V_2_ (7–26%), H_2_ (9%), L_2_ (9%), and I_2_ (10%), respectively, was possibly induced through intercalation of the nonpolar amino acids in the lipid layers without perturbing the initial phases to sufficient degree to induce a phase transition. On the other hand, arginine (174 g mol^−1^), different from other polar amino acids for its charged guanidinium group (—C—(NH_2_)_2_^+^), was shown to induce an MO phase transition from V_2_ to H_2_ at levels of >3.5% (w/v).^42^ With relevance to physiological contexts, some of the amino acids (such as alanine, arginine, glutamine, glycine) are typically supplemented at a total of 1% in cell culture media.^44^, ^45^, ^46^ Alternatively, l‐glutamine constitutes 0.01–0.06% of some ready‐to‐use mammalian cell culture media (e.g., in Medium 199 and Dulbecco's modified Eagle's medium, respectively), and up to 0.15% of endothelial cell growth media.^34^ Accordingly, the mesophase structure expansion or shrinking effects of the amino acids, as inferred from these studies, may be prominent if a certain threshold of lipid:amino acid (e.g., MO:arginine = 12:1^42^ or MO:tryptophan = 25:1^43^) is achieved in the media.

#### 2.1.3 Cell Culture Media

**Figure 2:**
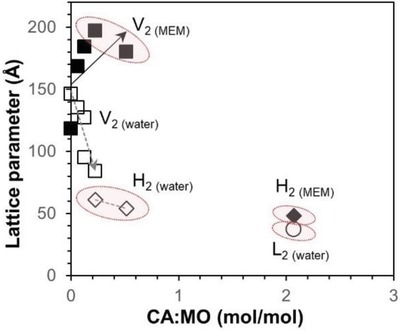
Phase behavior of monoolein‐capric acid (MO‐CA) nanoparticles (50 mg mL^−1^) stabilized by Pluronic F127 in either Milli‐Q water (open symbols: V_2_ □, H_2_ ◇, L_2_ ○) or in supplemented minimum essential media (MEM/10% FBS/1% nonessential amino acids, closed symbols: V_2_ ◾, H_2_ ♦) at 25 °C. Variations in the lipid phase behaviors are highlighted in the shaded areas. Adapted with permission.^44^ Copyright 2015, Royal Society of Chemistry.

Complexity of the composition of various cell culture media (as commercialized by Sigma, ATCC, and Life Technologies) makes it challenging to draw a general conclusion on the stability of LLC nanostructures when they come into contact with cell culture media. Only a limited number of studies have addressed the morphological stability of cubosomes in commonly used cell culture media. PHY cubosomes (both PEGylated and Plu‐F127 stabilized), when incubated in Dulbecco's modified Eagle's medium supplemented with 10% fetal bovine serum (DMEM/10% FBS), were shown to successfully retain their cubic structures with minute reductions in their lattice parameters within 24 h (without any significant difference at 25 and 37 °C).^47^ However, incubation for an extended period led to creaming of the cubosomes. In another binary system composed of MO doped with capric acid (CPP > 1) at varying molar ratio (i.e., CA:MO = 0–2.07), a dilution at 50 mg mL^−1^ in supplemented minimum essential media (MEM/10% FBS/1% nonessential amino acids) evidently suppressed the negative curvature effect of CA.^44^ Specifically, dispersion in MEM caused a noticeable increase in the lattice parameters (>20% increment); MEM also produced different phase behaviors than that in water especially at higher CA levels, where H_2_ in water appeared as V_2_ in MEM, and L_2_ in water existed as H_2_ in MEM (**Figure** **2**). Considering that the release kinetics are different across these nanostructures, such dissimilarities in the lattice sizes and phase behaviors critically point out a possible discrepancy and underestimation of drug release kinetics in simple buffer solutions as compared to that in cellular microenvironment. This highlights the need to examine the phase behaviors and drug release performance of LLC formulations using physiologically relevant media in order to establish a reliable in vitro–in vivo correlation.

#### 2.1.4 Blood Serum or Plasma

**Figure 3:**
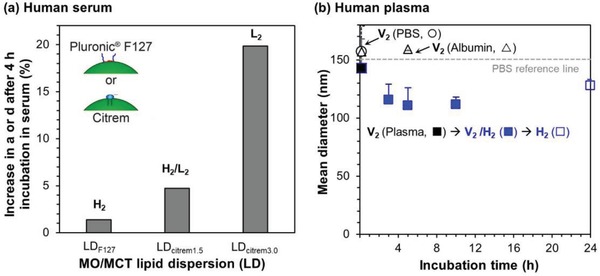
Modulatory effect of: a) human serum on the lattice parameter, *a*, and median diameter, *d*, of lipid dispersions consisting of monoolein and medium‐chain triglycerides (MO/MCT) in the presence of Plu‐F127 or citrem as a stabilizer. Reproduced with permission.^49^ Copyright 2015, Elsevier; b) human plasma on the particle size and phase behavior of a phytantriol‐based dispersion stabilized by Plu‐F127 (PHY‐F127). Adapted with permission.^50^ Copyright 2015, American Chemical Society.

Blood serum normally contains an abundance of albumin (≈60%), globulin (≈35%), fibrinogen (≈4%), and regulatory proteins, lipoproteins as well as iron‐binding proteins (≈1%).^48^ The adsorption affinity of these plasma proteins to LLC nanoparticles (i.e., the protein corona effects) is somewhat dependent on the choice of steric stabilizers and the lipid phases formed. For a system based on MO/medium‐chain triglyceride (MCT), Wibroe et al. compared the stabilization effect against serum using the anionic surfactant citrem and the nonionic amphiphilic polymer Plu‐F127.^49^ Similar to that observed in cell culture media,^44^ a short incubation in human serum (for 4 h at 37 °C) slightly increased the lattice parameter (*a*) and median diameter (*d*) of the binary dispersions stabilized by Plu‐F127 (LD_F127_) and that by citrem (LD_citrem_) (**Figure** **3**a).^49^ It was clearly shown that the L_2_ phase (LD_citrem3.0_) is more sensitive to the protein corona effects (with ≈20% size increment) than the corresponding H_2_ phase (LD_citrem1.5_, ≈5% increase). As speculated by the authors, this is possibly due to more efficient trapping of the serum proteins in the L_2_ “swollen” micellar aqueous cores in comparison with that of the H_2_ cylindrical nanochannels. Several other studies also reported the susceptibility of the MO components to esterase‐mediated hydrolysis, which resulted in phase destabilization and time‐dependent disintegration of the lipid nanoparticles in porcine and human plasma media.^50^, ^51^, ^52^, ^53^

On the other hand, the chemical structure of PHY does not possess an ester group, and it is therefore considered “nondigestible” from an esterase perspective, precluding an impact of chemical changes on self‐assembled structure when this PHY particle system is exposed to plasma. On contact of the indigestible PHY/F127 (10:1 w/w) system with human plasma (at 37 °C), the mean particle size was decreased by 9–29% relative to that in phosphate buffer, where the original V_2_^D^ phase started to diminish in 10 min and gradually evolved into a more negatively curved H_2_ phase within 17 h (Figure 3b).^50^ The authors ruled out the destabilizing effects of human serum albumin (which has an approximate ellipsoid dimensions of 3.8 × 3.8 × 14.0 nm^3^) on the dispersion particle sizes based on the fact that exposure to a physiological level of albumin (i.e., 35 mg mL^−1^) did not impart significant difference for 5 h. Nevertheless, Leesajakul et al. elucidated that albumin has three potential binding sites for MO molecules, where an increasing albumin level in the range of 2.8–15% induced higher degrees of MO extraction from the nanoparticles.^53^

Taken together, it appears that H_2_ phases may possess relatively higher stability against serum‐ or plasma‐induced size and phase modifications. It remains uncertain as to whether the current interfacial steric stabilization approach (e.g., using the Pluronics) is efficient in attenuating LLC interfacial interactions with plasma/serum components. The lack of interfacial shielding efficiency may in turn eliminate the desirable control over drug retention or release under biological environments.

### 2.2 Hematological and Immunological Responses

#### 2.2.1 Red Blood Cells

From the regulatory aspect, many of the nonlamellar forming amphiphilic lipids and stabilizers are generally recognized as safe (GRAS) for human external application and/or oral consumption. Information on the GRAS‐listed or Food and Drug Administration (FDA)‐approved excipients for drug formulations can be extracted via the FDA search engine “Inactive Ingredient Search for Approved Drug Products.” For example, MO and PHY are used as food additives as well as in skin and hair cosmetic products; Pluronic block copolymers, polysorbate 80, and citrem are commonly used emulsifiers, wetting agents, and dispersants.^45^, ^49^, ^54^, ^55^ When these individual components are formulated into complex nanostructure, their systemic “biocompatible window” and direct interactions with blood circulating cells are yet to be fully understood. **Table** **1** summarizes a number of findings addressing the hematological responses to MO‐ and PHY‐based nanoparticles, both of which are the most commonly studied LC lipids for drug delivery applications. Based on these short‐term hemolytic assays (via 1 h incubation), three major factors affecting the hemolytic propensity of LLC nanoparticles are identified: (i) type of lipid, (ii) lipid internal nanostructure, and (iii) feature of the polymer stabilizer.

**Table 1:**
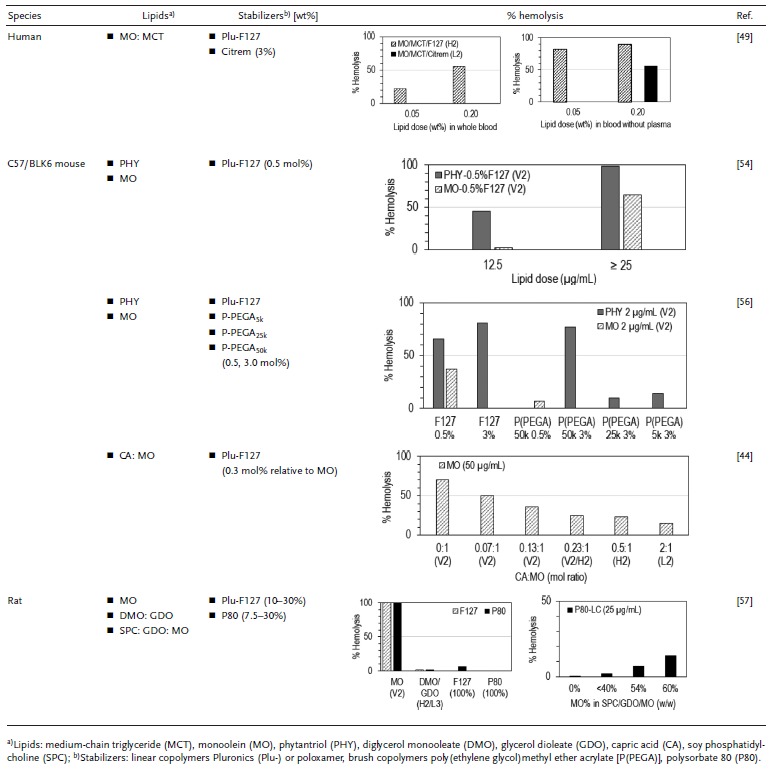
Short‐term in vitro hemolysis assay of liquid crystalline (LC) lipid nanoparticles in whole blood and isolated red blood cells (10^6^–10^7^ cells mL^−1^) from humans and rodents. All studies were conducted under static incubation (at 37 °C, 5% CO_2_) for 1 h where the LC lipids were dispersed in the presence of a stabilizer to form either inverse cubic (V_2_), inverse hexagonal (H_2_), sponge (L_3_), or other nonlamellar nanostructures. The amount of stabilizer is expressed as weight percentage (wt%) of the total lipid components unless otherwise specified. All data were reproduced with permissions.^49^ Copyright 2015, Elsevier;^54^ Copyright 2013, Royal Society of Chemistry;^56^ Copyright 2016, Royal Society of Chemistry;^44^ Copyright 2015, Royal Society of Chemistry;^57^ Copyright 2010, Elsevier

When comparing the type of lipid, it can be generalized that MO‐based cubosomes are relatively less hemolytic than PHY‐based cubosomes when formulated using the same type and amount of stabilizer. While the dose of PHY as low as 2–12.5 µg mL^−1^ was shown to induce a significant extent of lysis of red blood cells after 1 h exposure, MO‐based cubosomes have demonstrated almost 50% or less toxicity at the same doses.^54^, ^56^ The mesophase‐associated hemolytic effect of MO was further exemplified in a binary and a ternary system, each doped with capric acid (i.e., CA/MO),^44^ and soy phosphatidylcholine mixed with glycerol dioleate (i.e., MO/SPC/GDO),^57^ respectively. The incorporation of MO (CPP > 1) at increasing concentrations generally favors the formation of a V_2_ phase, which exerted relatively higher hemolytic effects than the corresponding H_2_, L_2_, and L_3_ phases.^44^, ^57^ Likewise, lipid dispersions in the form of H_2_ phase particles (comprised of MO/MCT/Plu‐F127) were more hemolytic than the equivalent L_2_ phase (MO/MCT/citrem), where the L_2_ phase particles could be tolerated at more than four times the concentration of the H_2_ phase particles.^49^ This collectively projects the trend that LLC nanoparticles with higher negative curvatures are less destructive toward cell membranes, presumably due to lower degrees of lipid mixing. Furthermore, endogenous plasma proteins may also offer significant protection against surface‐mediated hemolysis for all types of nanostructure; this was highlighted in a comparative analysis showing that removal of plasma from the human whole blood led to fourfolds and higher hemolysis for both H_2_ and L_2_ systems.^49^

Nevertheless, the hemolytic activities of dispersed mesophase particles can potentially be masked with interfacial coating using highly branched polymers, such as that demonstrated with the brush‐type copolymers consisting of multiple units of poly(ethylene glycol)methyl ether acrylate [P(PEGA)].^56^ Surface functionalization with P(PEGA), especially of the short and intermediate brush chain lengths (5–25 kDa), significantly reduced the hemolytic effect of PHY‐based cubosomes as compared to that with the linear copolymers Plu‐F127. The hematological safety profile was somewhat related to the lattice parameter of the resultant cubosomes in the order of P(PEGA)_25k_ (73.4 Å) > P(PEGA)_5k_ (71.5 Å) > Plu‐F127 (71.1 Å) > P(PEGA)_50k_ (69.8 Å). A larger internal channel diameter possibly implies a more efficient state of internalization of the hydrophilic arms of the stabilizer into the water nanochannels. Though not fully understood, Zhai et al. suggested that a balance between the external surface coverage and the internal incorporation of the stabilizing polymers is important toward manipulating the lipid transfer and biomembrane disrupting effects of LLC nanoparticles.

#### 2.2.2 Complement Activation and Proinflammatory Response

**Figure 4:**
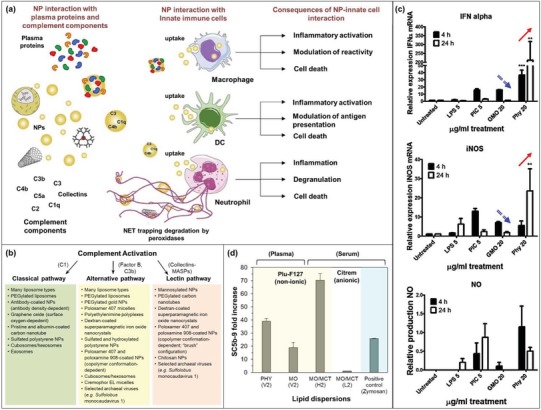
Immunological response of LLC nanoparticles: a) nanoparticle interaction with the innate immune system. Reproduced with permission.^58^ Copyright 2017, Elsevier; b) nanoparticle‐mediated complement activation through classical, alternative, and lectin pathways. Reproduced with permission.^58^ Copyright 2017, Elsevier; c) cell stress response of primary mouse splenocytes treated with 5 µg mL^−1^ lipopolysaccharide (LPS) or poly I:C (PIC), or 20 µg mL^−1^ MO or PHY cubosomes for 24 h (**p < 0.01, ***p < 0.001 compared to MO). Reproduced with permission.^54^ Copyright 2013, Royal Society of Chemistry; and d) effects of liquid crystalline lipid dispersions on plasma/serum level of the soluble form of membrane attack complex (SC5b‐9) as a measure of whole complement activation. Adapted with permissions.^49^, ^50^ Copyright 2015, Elsevier; and Copyright 2015, American Chemical Society, respectively.

The complement system, composed of at least 35 soluble and membrane‐bound proteins, serves as the first line of host defense system in response to systemic invasion of foreign particulate matters, such as pathogens and exogenous nanoparticles.^58^ The stimulation of biologically active complement peptides (e.g., C1, C3b, and collectins) inherently leads to uptake and phagocytosis of the opsonized nanoparticulate bodies by the immune cells (including macrophages, dendritic cells, and neutrophils) (**Figure** **4**a). One type of nanoparticle (may it be soft or rigid) potentially stimulates the complement cascade through more than one of these well‐known pathways: classical (e.g., liposomes, graphene oxide, and cubosomes/hexosomes), alternative (e.g., liposomes, PEGylated gold nanoparticles, dextran‐coated iron oxide nanocrystals, and cubosomes/hexosomes), and the lectin route (e.g., PEGylated carbon nanotubes, and dextran‐coated iron oxide nanocrystals) (Figure 4b).^58^, ^59^ Whether or not the subsequent inflammatory reactions propagate into life‐saving particle degradation and clearance, or detrimental homeostasis alteration and tissue damage, is an important subject of study for all injectable nanoparticles. To date, the collection of nanotoxicological studies have yet to provide a clear outlook on the acute adverse injection reactions as opposed to long‐term immunogenicity of LLC nanomaterials, especially on their repeated or continuous exposure. The internal use of LC lipids may become questionable if their systemic contact leads to severe anaphylactic reactions or inflammation, such as that reported for MO lipid in muscle and subcutaneous tissues even though the evidence remains anecdotal.^60^

It is noteworthy that systematic and detailed studies in the area of LLC immune response are rather lacking, with few studies limited to PHY‐ and MO‐based nanoparticles. Hinton et al. assessed the proinflammatory cell stress response in primary mouse splenocytes upon exposure to MO or PHY cubosomes (at 20 µg mL^−1^).^54^ As compared to the untreated control cells, PHY‐treated cells elicited progressive inflammatory response, where the expressions of interferon (IFN)‐α and inducible nitric oxide synthase (iNOS) were elevated, respectively, to 200‐folds and 23‐folds at 24 h (as indicated by the red/single‐lined arrows in Figure 4c). On the other hand, MO cubosomes provoked only a transient upregulation of IFN‐α (15‐folds) and iNOS (4‐folds) at 4 h but these markers resumed to the control levels by 24 h (as shown by the blue/double‐lined arrows in Figure 4c). This clearly shows that PHY nanoparticles are relatively more immunogenic than MO and even the positive controls including lipopolysaccharide and poly I:C. What is clear is that complement activation against formulation components such as PEG‐lipids in liposomes can dramatically alter the pharmacokinetics of particles on repeat administration, with significant consequences for their use in therapeutic applications.^61^ This aspect has not been studied at all to date for these nonlamellar systems.

Recently, a limited number of studies specifically addressed the immediate biochemical effects of PHY‐ and MO‐based nanoparticles on extracted human serum or plasma based on the expression of two end‐point complement markers: the early phase anaphylatoxin and chemoattractant agent, C5a, and the late phase soluble form of the membrane attack complex, SC5b‐9.^49^, ^50^ With a lipid dose fixed at 0.5 wt% in the plasma (note that this is significantly higher than the investigated hemolytic doses), LLC nanoparticles stabilized by Plu‐F127 activated both C5a and SC5b‐9 within 30 min of incubation at 37 °C. In particular, PHY (V_2_) elevated the level of SC5b‐9 by ≈39‐fold, whereas the MO (V_2_) counterpart appeared to elicit a milder immunogenic effect (i.e., a 19‐fold increase) (Figure 4d).^50^ When comparing the impact of internal nanostructure, MO/MCT‐based dispersions in the form of L_2_ phase (stabilized by citrem) appeared to be immunologically safer than the corresponding H_2_ phase (stabilized by Plu‐F127).^49^ In this study, the immunogenic effect of Plu‐F127 was excluded given that the dose applied (0.5 mg mL^−1^) was much lower than the immunogenic threshold (1.5 mg mL^−1^). Furthermore, the complement response remained unchanged when the concentration of Plu‐F127 was doped up to threefold higher in the MO/MCT‐based formulation. It was also confirmed that the anionic citrem did not block the calcium‐sensitive complement activation (i.e., the classical and lectin pathways) via chelation of the serum divalent cations, given that zymosan (a ligand prepared from yeast cell wall to induce experimental sterile inflammation) was still able to induce complement response in the presence of citrem‐stabilized formulations (Figure 4d, positive control). Wibroe et al. thus suggested the possible biological camouflaging role of citrem as rendered by its terminal citric acid moiety. Due to its structural similarity to that of sialic acid, citrem possibly shares the common strategy as adopted by some virulent bacterial pathogens in escaping the innate immune system via their surface expression of sialylated glycans and polysialic acid. Such findings suggest that more comprehensive screening of various stabilizers for their immune‐masking properties (especially across the arrays of different lipid type and nanostructure) may offer better strategies to optimize the immune safety of LLC nanoparticles. In addition, the effect of particle shape is eminently worth exploring in the design of LLC nanomaterials. This is supported by some recent findings showing that rod‐like or needle‐shaped nanoparticles have better negated the proinflammatory cytokine response and phagocytic uptake when compared to spherical nanostructures.^62^, ^63^, ^64^

### 2.3 Tissue Cytotoxicity

To foster the clinical translation of LLC nanoparticles, tissue biocompatibility has become an increasingly important subject of study over the last decade. Assessment of cell viability using the colorimetric Alamar Blue or MTT [3‐(4,5‐dimethylthiazol‐2‐yl)‐2,5‐diphenyl‐2*H*‐tetrazolium bromide] assay is a common metabolic indicator to measure the tolerance of mammalian cells toward these exotic nanostructured particles. **Table** **2** summarizes the collection of short‐term in vitro cytotoxicity studies (1–72 h) of LLC nanoparticles (with average hydrodynamic diameters in the range of 100–400 nm) in isolated animal and human cells. Case studies are presented below (in accordance to the designated case numbers in Table 2) to highlight some key strategies utilized to manipulate the cytotoxicity profiles of LLC nanoparticles. These include the incorporation of naturally occurring phospholipids, effective surface coverage using different forms of stabilizers, and surface charge modification. All these processes may result in a change in the lattice parameter and/or the mesophase behavior. Herein, we intend to clarify if there is a correlation between the internal nanostructure or surface curvature of LLCs and their cellular toxicity or uptake profiles. Cellular toxicity is typically reflected by the half or 80% maximal inhibitory concentration (IC_50_ or IC_80_), where lower IC values indicate higher toxicity. Alternatively, the percentage of cell viability is quantified against the untreated control group at a fixed lipid dose.

**Table 2:**
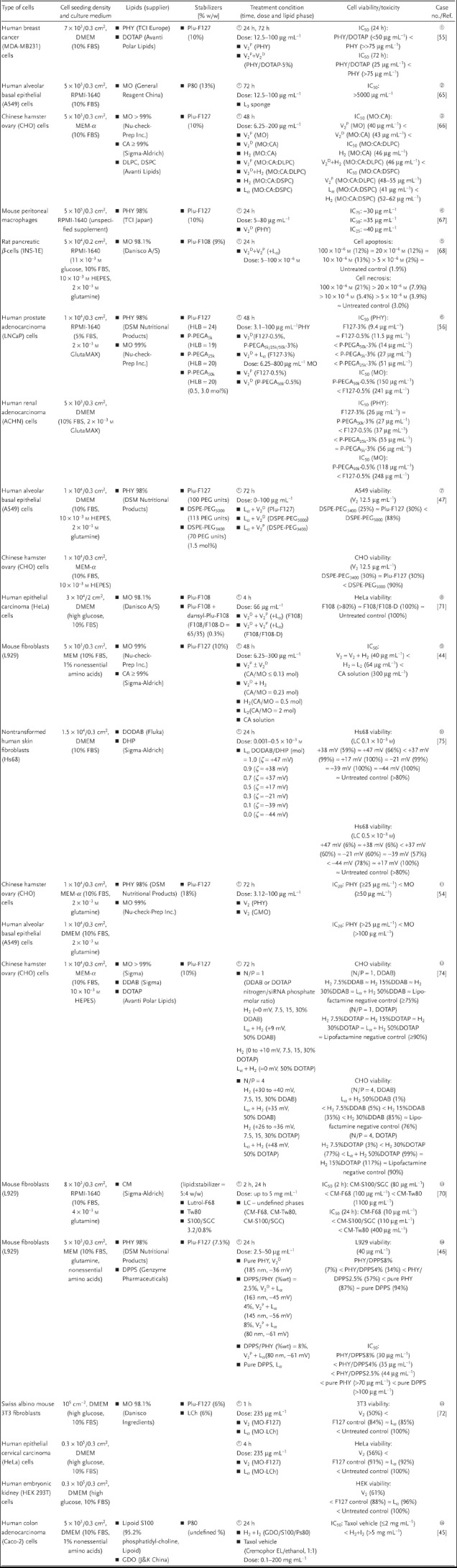
Short‐term in vitro cytotoxicity assay of liquid crystalline (LC) lipid nanoparticles in isolated animal and human cells. All studies reported were conducted on static, planar cell culture models (incubated at 37 °C, 5% CO_2_) where the lipids were dispersed in the presence of a stabilizer to form either inverse cubic (V_2_), inverse hexagonal (H_2_), reversed micellar cubic (I_2_), inverse micellar (L_2_), or lamellar (L_α_) nanostructures. The amount of stabilizer is expressed as percentage weight relative to the lipid (wt%) unless otherwise specified. Lipids: phytantriol (PHY), monoolein (MO), diglycerol monooleate (DMO), glycerol dioleate (GDO), 1,2‐dioleoyl‐3‐trimethylammonium‐propane (DOTAP), capric acid (CA), 1,2‐dilauroyl‐*sn*‐glycero‐3‐phosphocholine (DLPC), 1,2‐distearoyl‐*sn*‐glycero‐3‐phosphocholine (DSPC), cholesteryl myristate (CM), dioctadecyldimethylammonium bromide (DODAB), dihexadecyl phosphate (DHP), dipalmitoyl phosphatidylserine (DPPS), didodecyldimethylammonium bromide (DDAB); Stabilizers: linear triblock copolymers Pluronics (Plu‐) or poloxamer, brush copolymers poly(ethylene glycol)methyl ether acrylate [P(PEGA)], Tween 80 (Tw80), or polysorbate 80 (P80), 1,2‐distearoyl‐*sn*‐glycero‐3‐phosphoethanolamine‐N‐[methoxy(poly(ethylene glycol)] (DSPE‐PEG), blend of soy phospholipids (Lipoid S100) and sodium glycocholate (S100/SGC), and lauroylcholine chloride (LCh); Culture media: Roswell Park Memorial Institute medium (RMPI‐1640), fetal bovine serum (FBS), Dulbecco's modified Eagle's medium (DMEM), minimum essential medium α (MEM‐α); Toxicity indicators: half maximal inhibitory concentration (IC_50_) where lower IC_50_ values indicate higher toxicity

#### 2.3.1 Incorporation of Phospholipids

Based on an 80% cell viability threshold, cubosomes composed of digestible MO could be tolerated at twice as much the dose of indigestible PHY‐based counterparts in hamster ovary (CHO) cells (i.e., 50 µg mL^−1^ vs 25 µg mL^−1^), or higher in human alveolar cancer (A549) cells (≈100 µg mL^−1^ vs ≈25 µg mL^−1^) (case ).^54^ This is comparable to the trend in their hemolytic activities albeit the magnitude of tolerable lipid dose is much lower in red blood cells. Both MO‐ and PHY‐based cubosomes (at 20 µg mL^−1^) significantly increased the apoptotic markers (BAX/BCL2 ratio) and oxidative stress related genes (heme oxygenase‐1 and gluthathione reductase) within 24 h.

Nevertheless, the safety window of MO or PHY is tunable by incorporating certain endogenous phospholipids as a closer mimic of the major constituents of mammalian cell membranes (i.e., ≈60% phospholipids). Examples include capric acid (CA) and phosphatidylcholines (e.g., DLPC and DSPC).^44^, ^66^ CA (with a CPP > 1) was incorporated at increasing amounts to induce phase transformation of MO dispersions from V_2_^P^ → V_2_^D^ → H_2_ → L_2_ (case ⑨).^44^ Pure CA solution was confirmed to induce subtle alteration to the viability of fibroblast cells (L929) at concentrations as high as 300 µg mL^−1^. The tolerability ranking was followed by the more negatively curved H_2_ and L_2_ dispersions, which displayed better safety with an IC_50_ value 35% higher than that of the V_2_‐containing formulations (i.e., 64 µg mL^−1^ cf. 40 µg mL^−1^). Interestingly, phase stabilization of MO/CA systems in the form of V_2_ and H_2_ (but not L_α_) by incorporating DLPC and DSPC, respectively, improved the tolerable doses of the resultant ternary systems (case ③). Specifically, the IC_50_ of cubosomes was increased from 43 µg mL^−1^ (MO/CA) → 48–55 µg mL^−1^ (MO/CA/DLPC), and that of hexosomes was enhanced from 46 µg mL^−1^ (MO/CA) → 52–62 µg mL^−1^ (MO/CA/DSPC).^66^ An important implication is that, doping with a higher endogenous phosphatidylcholine content may contribute to stabilizing the desired cubic or hexagonal phases and simultaneously reducing the cytotoxicity effects. According to their intrinsic nature, phosphatidylcholine typically resides in the outer leaflet of cell membrane to confer a permeability barrier. It is therefore plausible that the mobility of the host lipids and their cellular interactions were more restricted in the presence of DLPC (C12:0) and DSPC (C18:0).

On the contrary, negatively charged phosphatidylserine (e.g., DPPS), which is naturally located in the inner leaflet of cell membrane but is capable to migrate to the outer leaflet during apoptotic distress, was shown to exaggerate the fusogenic activity of PHY‐based dispersions (case ). While pure DPPS vesicles (up to 100 µg mL^−1^) and PHY cubosomes alone (at 40 µg mL^−1^) did not induce significant cell death (i.e., L929 fibroblasts viability > 85%), the incorporation of DPPS into PHY at increasing concentrations (within 2.5–8 wt%) resulted in 34–91% reduced viability relative to pure DPPS.^46^ This is accompanied by a V_2_ → V_2_/L_α_ phase transformation. Taken together, it seems that a phase transformation favoring the lamellar behavior (i.e., L_α_ vesicles) correlates with an increase in cellular uptake and cytotoxicity. While the authors neglected the importance of the phospholipid biofunctions, we emphasized that the intrinsic nature of the selected phospholipids in cell membranes needs to be recognized as this may be related to their positive or negative impact on the fusogenic and cytotoxicity potential of LLC formulations.

#### 2.3.2 Surface Coverage with Stabilizers

The Pluronic series of linear, triblock PEO‐PPO‐PEO copolymers (particularly F127) have served as a gold standard in LLC stabilization and “stealth” functionalization, to which other steric polymers are often compared with. From the aspect of colloidal stability, Chong et al. have previously verified the dependency of the stabilizing power of Pluronics on their: (i) PEO hydrophilic chain lengths (i.e., the PEO layer needs to be at least 19–20 repeat units long, equivalent to ≈25 Å, to confer stable dispersions); (ii) PPO hydrophobic domain (e.g., F108 with 50 PPO units preserved the cubic phase integrity of MO better than F127 with longer PPO units of 65); (iii) hydrophilic–lipophilic balance (where HLB ≥ 23 creates an efficient dispersion); and (iv) molecular weight (e.g., F108 with a higher MW of 14 600 formed more stable dispersions than F68 with a lower MW of 8400, regardless of their similar HLB).^69^ In terms of biocompatibility, more evidence‐based data are necessary to accurately define the cellular protective and stealthy functions of these molecules. Fortunately, most steric stabilizers or additives alone are relatively inert toward cell lysis or metabolic alteration. However, the choice of stabilizers other than the conventional Pluronic polymers can impart significant influences on the safety window of various LLC nanostructures. Examples include those demonstrated with Tween 80 (an amphiphile with single alkyl chain),^70^ DSPE‐PEG (PEGylated phospholipids with multiple alkyl chains),^47^ and P(PEGA) (nonlinear brush‐type copolymers).^56^

Tween 80 (CPP << 1) was shown to enhance the tolerability of an MO dispersion (in the less negatively curved sponge phase), which displayed a relatively high IC_50_ > 5000 µg mL^−1^ in A549 alveolar cells after 72 h of exposure (case ②).^65^ The MO/Tween 80 spongosomes also boosted the in vitro antiproliferative efficacy of a water‐insoluble traditional medicine, *Brucea javanica* seed oil (BJO). The mechanism was attributed to enhanced BJO dispersion in a sustained release manner from the lipid matrix, which resulted in 1.8–2.4 times lower IC_50_ dose than that of pure BJO in inhibiting the tumor cell growth. The sponge phase is considerably more biocompatible than the MO/Plu127 cubosomes, which showed an IC_50_ in the range of 40–250 µg mL^−1^ in various cell lines.^44^, ^54^, ^56^, ^66^, ^71^, ^72^ In another 24 h cytotoxicity test using L929 fibroblasts, an LC dispersion (5% cholesteryl myristate and 4% stabilizer, w/w) stabilized by F68 was rather poorly tolerated in comparison with the counterparts stabilized by soy phospholipids (S100)/sodium glycocholate (SGC), and that by Tween 80 (case ). The IC_50_ values increased in the order of: CM‐F68 (10 µg mL^−1^) < CM‐S100/SGC (110 µg mL^−1^) < CM‐Tw80 (400 µg mL^−1^), emphasizing the protective capability of Tw80, although the lyotropic liquid crystalline phase behavior was not clarified in the study.^70^

As realized from the hematological safety profile (Table 1), the cytotoxicity of LLC nanoparticles can be modulated via effective surface coverage and controlled internal insertion of the stabilizer. Zhai et al. examined the cytotoxicity trend of PHY‐based cubosomes stabilized with Plu‐F127 (possessing 2 × 100 PEG units, with a hydrophobic PPO segment in the middle) in comparison with three types of PEGylated phospholipids, specifically DSPE conjugated with PEG of 2000, 3400, and 5000 Da (corresponding to 45, 70, and 113 PEG units, respectively).^47^ A 72 h exposure to a fixed PHY dose (12.5 µg mL^−1^) showed a clear trend that surface coverage using the longer chain DSPE‐PEG_5000_ conferred superior cell viability (≥88%) than that with the shorter chain DSPE‐PEG or Plu‐F127 (≈30%) (case ⑦). DSPE‐PEG_5000_ also preserved the integrity of the original *Pn3m* (V_2_^D^) crystallographic space groups with unchanged lattice parameters (with reference to the bulk PHY). An explanation is that DSPE‐PEG_5000_, owing to its bulkier chain and limited insertion into the internal matrix of PHY nanoparticles, favorably resides on the nanoparticle surface to impart a higher density of steric corona. This inherently creates a greater barrier to nanoparticle–cell interaction or material exchange. In contrast, Plu‐F127 induced the formation of liposomes more than cubosomes, in which the surface availability of the steric PEG chains is attenuated. DSPE‐PEG of shorter chains were also postulated to present fewer PEG units at the surface since they caused noticeable swelling of the nanoparticles via internal incorporation, as such were unsuccessful in improving the cytotoxicity profiles as compared to the control Plu‐F127 cubosomes. However, such an inverse relationship between the hydrophilic chain length and nanoparticle cytotoxicity is not as obvious in the case of the nonlinear, brush‐type copolymers [P(PEGA)].^56^ As revealed by the hemolytic profile, the intermediate chain [P(PEGA)_25k_] also displayed significantly better cellular tolerance than the longer chain [P(PEGA)_50k_] and Plu‐F127 via partial entrapment into the internal and external microenvironment of the nanoparticle (case ⑥). This may have an impact on the lipid mobility and material transfer albeit by unclear mechanisms. A better understanding of the exact mechanisms behind the surface coverage and internalization of various stabilizers is thus an important step to establish their structure–function relationships to better design a biocompatible LLC formulation.

#### 2.3.3 Surface Charge Modification with Cationic Moieties

Due to the net negative surface charge of mammalian cell membranes, cationic vectors are frequently investigated as a “Trojan horse” to enhance intracellular trafficking of therapeutic molecules endowed with difficult membrane permeability. Naturally, phagocytic cells (such as macrophages) preferentially take up anionic nanoparticles (presumably resembling the bacteria) whereas nonphagocytic cells (e.g., epithelial cells) interact more strongly with cationic nanoparticles.^73^ While cationic nanoparticles typically induce a transient disruption to the integrity of the cell membrane during internalization, whether or not this leads to compromised cell viability (i.e., necrotic death) in addition to the increased cargo uptake is a major concern for clinical applicability. For an MO matrix doped with a short‐chain cationic surfactant, lauroylcholine chloride (LCh), a phase transition of V_2_ → L_α_ is prompted.^72^ These two dispersion forms were tested for their cytotoxicity in three different cell lines, including mouse fibroblasts 3T3, human cervical cells HeLa, and human renal cells HEK 293T (case ). The excellent tolerability of the cationic liposomes (MO/LCh) is strongly corroborated by the well‐preserved viability across all these cell lines at an MO dose of 235 µg mL^−1^ (i.e., 85–96%) as opposed to 39–50% of cell death caused by the conventional negatively charged cubosomes (MO/F127).

Nevertheless, the formation of a lamellar phase does not always lead to a safer profile as shown in other anionic or cationic systems.^47^, ^56^, ^66^ The resultant charge ratio in conjunction with the type of cationic dopant appeared significant in influencing cytotoxicity.^74^, ^75^ In one particular case where MO was combined with cationic lipids complexed with siRNA (short interfering ribonucleic acid), an increasing concentration (7.5–50 wt%) of DDAB (didodecyldimethylammonium bromide) or DOTAP (1,2‐dioleoyl‐3‐trimethylammonium‐propane) triggered the transformation of H_2_ → L_α_/H_2_ mixed phase (case ). At neutral or low positive charge (0 to +10 mV), all systems were well tolerated in CHO ovary cells regardless of the nanostructure and cationic lipid type. When the surface charge was elevated by reducing the siRNA/lipid ratios (+26 to +48 mV), DDAB‐based L_α_/H_2_ structure exerted much higher cytotoxicity than its H_2_ analogues (i.e., 1% vs 5–85% viability, respectively). Judging from the internal nanostructure, the nonlamellar matrices (H_2_) mostly produced superior cell viability as well as efficacy in siRNA gene silencing than their lamellar analogues (L_α_). However, such lamellar phase‐induced cytotoxicity was absent when DDAB was replaced with DOTAP in spite of their comparable high positive surface charges. These findings inferred that the magnitude of surface charge (i.e., low is better than high positive) together with the type of cationic dopant (in this case, DOTAP is more tolerable than DDAB) are the major determinants of LLC cytotoxicity. However, the enhanced tolerability by doping with the “relatively safer” DOTAP was not manifested in another study, where PHY/DOTAP cubosomes (+37 mV) appeared to be more cytotoxic than the original PHY cubosomes (−34 mV) toward human breast cancer cells MDA‐MB231 (IC_50_ = 25 µg mL^−1^ vs >75 µg mL^−1^, respectively, in case ①).^55^ Interestingly, partial charge neutralization using cationic DODAB (dioctadecyldimethylammonium bromide)/anionic DHP (dihexadecyl phosphate) was proved to be an effective strategy to generate cationic liposomes (+17 mV) that are nontoxic to human skin fibroblasts Hs68 in comparison with the corresponding high‐positive or high‐negative vesicles (case ).^75^ Not all these observations reached a clear consensus; therefore, it is challenging to outline a simple rule regarding the charge‐dependent cytotoxicity of LLC systems. There is a need to untangle the complex interplay between the nanostructure, cationic lipid type, and the overall particle charge in predicting the tissue tolerability of LLC nanoparticles.

## 3 Cellular Uptake and Lipid Transfer Mechanisms

### 3.1 Mechanisms of Uptake and Intracellular Distribution

Mechanistic studies of cellular uptake and intracellular fate of nanoparticles, mostly associated with the context of drug delivery efficiency, are often overlooked for their relevance to cytotoxicity. In fact, whether or not the LC lipids translocate across the cell membrane to reach the cytosol and internal compartments (such as endosomes), or cause disruption to the nuclei membrane, would inherently have an impact on the cell viability. Generally, cellular uptake of nanoparticles may occur via specific pathways including: (i) phagocytosis by specialized phagocytes (especially for those larger than 0.5 µm) or (ii) protein‐coat‐driven endocytosis (either clathrin‐ or caveolin‐mediated), as well as nonspecific pathways such as (iii) macropinocytosis that utilizes membrane lipids to drive large vacuole formation (0.2–0.5 µm), or (iv) pinocytosis involving small pinocytic vesicles (≈100 nm) for “cellular drinking.”^76^ A combination of factors such as the particle size, shape, surface chemistry, as well as viscoelasticity contribute to controlling the cellular uptake mechanisms and intracellular fate, which may in turn dictate the cytotoxicity.^76^, ^77^ The viscoelastic behaviors of LLC mesophases arising from phospholipid‐based foods and formulation materials can be identified as purely plastic (lamellar), viscous/fluid (hexagonal, ≈10^1^ kPa), or elastic/solid (cubic, ≈10^3^ kPa).^77^, ^78^, ^79^ Using hydrogel particles as a model, it was shown that “soft” nanoparticles (<35 kPa) are prone to macropinocytosis (and therefore, undergoing faster cellular uptake) while “stiff” nanoparticles are mostly internalized via multiple entry mechanisms, especially the clathrin‐ and caveolae‐mediated endocytosis in addition to macropinocytosis (and hence, a time‐dependent uptake).^80^ On this basis, characterization of the rheological behaviors alongside morphological properties of an LC mesophase may enable prediction toward their uptake mechanisms and thus, intracellular fate. Presented below are some specific studies determining the cellular uptake pathways of various LLC nanoparticles using the well‐established biochemical approaches.

**Figure 5:**
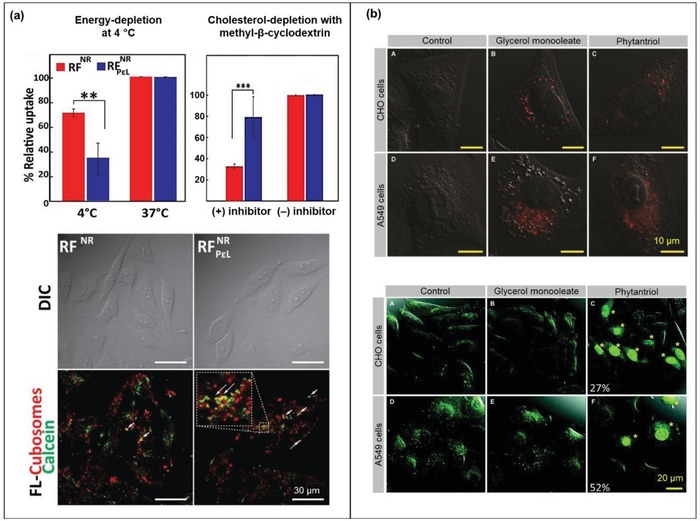
Cellular internalization and distribution patterns of LLC nanoparticles: a) uptake of Nile red‐labeled MO/Plu‐F127 cubosomes with (RF_NR/P_ *_ε_*_L_) or without (RF_NR_) poly‐ε‐lysine coating by HeLa cells after 1 h of incubation (**p < 0.01 and ***p < 0.001). In the confocal images, green spots refer to the calcein‐stained endosomes; red spots indicate the Nile red lipid probe. Reproduced with permission.^81^ Copyright 2017, American Chemical Society; and b) uptake of Rhodamine R18‐labeled MO or PHY cubosomes (red, top figures) and Lucifer yellow (green, bottom figures) by CHO and A549 cells at 24 h (asterisks mark cells with disrupted membrane and nuclei staining). Reproduced with permission.^54^ Copyright 2013, Royal Society of Chemistry.

Zeng et al. characterized the principle mechanisms of LLC nanoparticle uptake by treating the human intestinal Caco‐2 cells with selective endocytosis inhibitors prior to quantifying their internalization degree: ATP energy‐dependent pathway (blocked by NaN_3_/2‐deoxyglucose), cholesterol raft‐dependent endocytosis (by methyl‐β‐cyclodextrin), caveolae‐mediated endocytosis (by filipin), clathrin‐mediated pathway (by chlorpromazine), and actin‐dependent uptake (by cytochalasin D).^45^ The uptake of a mixed H_2_/I_2_ dispersion composed of glycerol dioleate (GDO)/soy phospholipid/polysorbate 80 occurred largely via the classical energy‐dependent clathrin‐mediated pathway, in conjunction with the caveolae‐ and noncaveolar lipid raft‐mediated routes. Interestingly, Deshpande and Singh further verified that the conventional MO/Plu‐F127 cubosomes (with a net surface charge of −20 mV) were also able to enter different cell lines (including human cervical HeLa cells, murine fibroblast NIH 3T3 cells, and human breast cancer MDA‐MB231 cells) via energy‐independent translocation (**Figure** **5**a, RF_NR_).^81^ Further surface charge modification with poly‐ε‐lysine (a cationic polymer conferring neutral cubosomes of +1 mV) caused the nanoparticles to rely strongly on the energy‐dependent, clathrin‐mediated endocytosis but less susceptible to the energy‐independent and cholesterol‐mediated uptake mechanisms (Figure 5a, RF_NR/PεL_).

With respect to intracellular distribution, the GDO‐based H_2_/I_2_ and MO‐based V_2_ nanoparticles were primarily located within the membrane‐enclosed vesicular regions (i.e., endosomes or lysosomes) as visualized by their discrete punctate staining pattern outside the nuclei (confocal images in Figure 5a).^45^, ^54^, ^82^ Entrapment in the endocytic vesicles was further increased for the poly‐ε‐lysine coated MO (V_2_) nanoparticles as shown by greater colocalization of the Nile red‐tagged MO and the calcein‐stained endosomes. This is different from the PHY (V_2_) nanoparticles that exhibited a rather diffuse cytoplasmic staining pattern, indicating that either PHY was freely migrating within the cytoplasm or there was some membrane mixing of the cubosome‐dye components (Figure 5b, top figures).^54^ Coformulation of PHY with the anionic, fusogenic phospholipid DPPS also enhanced the cytoplasmic distribution of the cubosome‐dye.^46^ In almost most cases, these LLC nanoparticles were unable to translocate across the nuclei membrane. Yet, PHY cubosomes (but not MO) significantly disrupted the nuclear membrane integrity as evidenced by the subsequent nuclei staining with Lucifer yellow (that only passes through disrupted membranes) (Figure 5b, bottom figures). This can be linked to the generally higher hemolysis and cytotoxicity behaviors of PHY‐based nanoparticles as formerly discussed. Furthermore, cellular treatment with PHY nanoparticles for 45 min also led to subsequent inhibition of the macropinocytosis pathway, where the cellular uptake of the specific marker (dextran) was markedly reduced presumably attributed to disrupted plasma membrane turnover.^54^ However, the clathrin‐mediated route remained robust as verified by the unmodified selective uptake of transferrin across the PHY‐treated and untreated control cells. The fact that MO nanoparticles have left the specific/nonspecific uptake pathways unaffected and the nuclei membrane integrity intact suggests that it can be a relatively safer LC lipid suitable for clinical use. However, the greater endosomal entrapment (particularly those entered via the clathrin‐mediated endocytosis) also conferred a higher chance of enzymatic degradation of the nanoparticulate body, which is a major downside if cytosolic delivery of an intact particle is desired as in most of the cases. In this regard, internalization via the lipid raft‐guided uptake and caveolar endocytosis are of greater interests to selectively drive cargo delivery into the cell cytosol since these routes are unlikely to fuse with lysosomes and thereby, promoting escape from the lysosomal degradation.^83^, ^84^

### 3.2 Mechanisms of Membrane Fusion and Lipid Transfer

**Figure 6:**
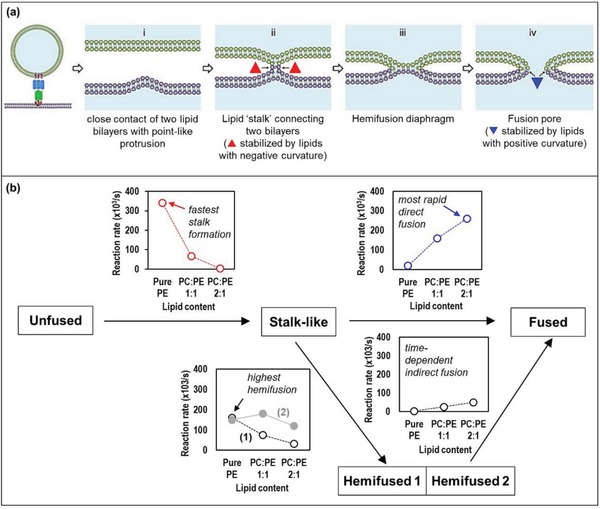
Membrane fusion mechanisms: a) schematic diagram illustrating different steps of membrane fusion in the stalk‐pore model with homogenous membranes. Reproduced with permission.^88^ Copyright 2016, Nature Publishing Group; b) reaction rates simulated for each reaction pathway during fusion of lipid vesicles consisted of palmitoyloleoyl phosphatidylcholine (POPC) and palmitoyloleoyl phosphatidylethanolamine (POPE): pure POPE, POPC:POPE = 1:1, and POPC:POPE = 2:1. Adapted with permission.^87^ Copyright 2007, Public Library of Science.

The fusogenic propensity of LC lipids is expected to associate with their hemolytic and tissue cytotoxic properties due to the ability to alter the plasma membrane compositions and hence, the biophysical qualities. Examples of amphiphilic molecules that display strong fusogenic character include MO, oleic acid, and palmitoleic acid; GDO, methyl stearate, and methyl palmitate are considered weakly fusogenic, whereas glyceryl monostearate and glyceryl monopalmitate are nonfusogenic.^85^ Intrinsically, the coexistence of “bilayer forming” and “nonlamellar forming” glycerolipids in biological membranes (e.g., the respective couple PC/PE in yeast and animal cells, and PG/PE in *Escherichia coli*) do play a crucial role in regulating membrane fusion and therefore, interactions with lipid nanoparticles even in the absence of fusion proteins.^86^ In a computational molecular dynamics simulation of lipid vesicle fusion, Kasson and Pande predicted that the underlying pathways of membrane fusion differ remarkably with lipid compositions.^87^ A “branching fusion pathway” was illustrated, which involved an initial formation of a contact “stalk‐like intermediate” between two adjacent lipid vesicles (this is in line with several other membrane fusion studies describing the “stalk‐pore model”^86^, ^88^) (**Figure** **6**a). Subsequently, the lipid stalks might undergo (i) a direct and rapid fusion via a coordinated change in both inner and outer leaflets, or (ii) an indirect fusion via a metastable “hemifused intermediate” on a microsecond timescale. The simplistic simulations revealed that vesicles composed of pure POPE (palmitoyloleoyl phosphatidylethanolamine, a “nonlamellar forming” lipid) fused predominantly via an indirect hemifused intermediate (Figure 6b). On the contrary, those composed of mixed POPC (palmitoyloleoyl phosphatidylcholine, “bilayer forming”) and POPE (2:1 w/w) fused directly from the stalk structure with only 11% undergoing the indirect hemifusion mechanism. The POPC:POPE lipid mixtures, having closer resemblance to the physiological membrane compositions, possibly reflect the major fusion mechanism being the direct pathway. However, the role of specialized fusion proteins and the nonuniformity in membrane components may further complicate the exact underlying mechanisms. In addition to lipid compositions, membrane curvature also imparts significant influence on the rate of fusion. The stalk formation was shown to be faster in 15 nm vesicles than in 19 nm vesicles, presumably due to the higher membrane stored curvature elastic energy associated with the higher curvature.^86^, ^87^, ^89^

In addition to theoretical predictions, the process of membrane fusion has been elucidated by various interfacial acoustic sensing and diffraction methods. Shen et al. confirmed that the presence of a naturally occurring anionic phospholipid, DPPS, enhances the fusogenic potential of PHY nanoparticles, which is in correlation with the higher in vitro cytotoxicity profile.^46^ Specifically, they studied the dynamic surface behavior of a PHY/DPPS anionic dispersion toward a supported bilayer model composed of 1‐palmitoyl‐2‐oleoyl‐sn‐glycero‐3‐phosphatidylcholine (POPC) using quartz crystal microbalance with dissipation monitoring (QCM‐D). PHY/DPPS‐2.5% with a mixed phase of V_2_^D^/L_α_ (−45.2 mV) underwent a higher rate of lipid molecular exchange and a greater final mass uptake by the model bilayer in comparison with the unmodified PHY cubosomes (V_2_^D^, −36.4 mV). While there is a minute difference in the surface charges, the increased fusogenicity of PHY/DPPS‐2.5% was likely to be contributed by the more hydrophobic lamellar structures, where their surface coverage with Plu‐F127 was depleted. The authors gained further insight into the PHY cubosome structural changes during the mixing process in solution with POPC “membrane‐like” vesicles. SAXS profiles show an increase in the cubic phase lattice sizes (presumably due to POPC insertion into the cubic phase lattice) accompanied by a structural rearrangement tending toward the lamellar phase at the expense of the cubic phase. This is in qualitative agreement with the findings of Vandoolaeghe et al. that revealed a tentative transition of V_2_ → V_2_/L_α_ following a progressive lipid exchange between MO/Plu‐F127 nanoparticles and the surface layer of a dioleoylphosphatidylcholine (DOPC) bilayer membrane.^90^ As determined using neutron reflectivity, the DOPC bilayer was altered from 80% DOPC surface coverage to 8%:72% of DOPC:MO mixed surface at the steady state (≥18 h).

Intriguingly, how these mechanisms and kinetics of fusion as well as the compositional balance between nonlamellar‐ and bilayer‐forming lipids correlate with the endogenous/exogenous lipid exchange have remained to be clearly elucidated. An enhanced understanding of the patterns of lipid uptake and exchange between various LC lipids and biological membrane is expected to create an important indicator for their hemolytic and tissue cytotoxicity effects.

## 4 In Vivo and Clinical Safety Aspects of LLC Materials

In living systems, membranes with cubic or hexagonal periodicity do exist across all kingdoms of life (ranging from protozoa and yeasts to mammals) that possibly evolve in a reversible manner from any cytomembrane, such as the plasma membrane, endoplasmic reticulum, nuclear envelope, mitochondrial membrane, and the Golgi complex.^86^, ^91^ The major differences between natural cubic membranes and fabricated cubic phases lie in their unit cell size and water activity.^91^ Simple cubic systems based on lipid/water usually have a lattice size of 20 nm or less, which is significantly smaller than that of the cellular cubic membranes ranging in the scale of 50–500 nm. While fabricated cubic phases typically exhibit extremely high water activity (70–90%), the arrangement of cubic membrane in vivo is involved in osmolarity control, curvature‐regulated enzyme activity, synaptic transport, and photonic function (such as that occurred in bioluminescent scaleworms resulting from their different cubic membrane subtypes). Interestingly, natural membranes do rely on the formation of hexagonal structures in carrying out certain enzymatic activities (e.g., calcium pump in the sarcoplasmic reticulum, and the violaxanthin de‐epoxidase in the thylakoids) as well as the membrane anchoring of some proteins (such as the G‐proteins and phosphokinases C).^86^, ^92^, ^93^, ^94^, ^95^ It has also been illustrated that cubic biomembranes may act as “shelter” to protect biologically essential macromolecules (such as RNAs) from oxidative damage during protein translation.^96^ The fact that appearances of “unusual cubic membranes” are closely associated with pathological cellular states (e.g., viral infections) and food depleted conditions (e.g., in starved amoeba *Chaos*) suggests an intriguing possibility of cubic structure–cellular stress relationships.^91^, ^96^

To date, there is still lack of documentation on the short‐ and long‐term biosafety of nanostructured LLC systems. A myriad of in vivo investigations have mostly focused on the biopharmaceutical performances of LLC materials in terms of improved oral or topical drug pharmacokinetics;^4^, ^45^, ^97^, ^98^, ^99^, ^100^ controlled subcutaneous therapeutics via stimuli‐responsive release;^5^, ^101^, ^102^ as well as enhanced vaccination via systemic potentiation of immunogenic responses.^67^ Biosafety profiling of these exotic LLC nanoparticles, which is of paramount importance to their clinical translation, seems to be either underexplored or undisclosed. In fact, direct analysis of the biodistribution of LLC nanoparticles following administration is rarely reported, possibly complicated by their unknown degradation pathways and chemical identities that overlap with that of the endogenous cellular lipids. Nevertheless, the biofate of LLC nanoparticles could be inferred from the tissue distribution of the encapsulated model compounds (based on the assumption that partition of the drug‐carrier does not occur). This is exemplified by the study of Lee et al., where the in vivo tissue distribution of a hydrophobic peptide‐based drug candidate (i.e., a new hepatitis C virus NS5A inhibitor, BMK‐20113) was evaluated in Sprague‐Dawley rats for a lamellar phase LLC formulation in comparison with a nonlipid cyclodextrin formulation.^28^ Following an intravenous bolus dose, the lamellar phase liquid crystalline nanoparticles (formulated using Emulgrade SE‐PF/PEG‐12 cetostearyl ether/tetradecyl tetradecanoate, with average vesicle sizes of 70–130 nm) showed the highest retention in the liver as compared to other tissues in the following sequence: liver (51.5 µmol g^−1^) > kidney (29.6 µmol g^−1^) > lung (16.4 µmol g^−1^) > blood plasma (9.4 µmol g^−1^). This is different from the accumulation pattern of the nonlipid cyclodextrin formulation: kidney (48.4 µmol g^−1^) and plasma (38.6 µmol g^−1^) > lung (29.3 µmol g^−1^) > liver (18.9 µmol g^−1^). Interestingly, administration via the oral route showed similar trend of tissue distribution for both formulations. While the tissue toxicity was not assessed, Lee et al. regarded that the lamellar phase LLC nanoparticles can potentially act as a liver‐specific drug delivery system. Further hepatotoxicity and clearance studies are clearly warranted to justify the internal applicability of the LLC nanoparticles.

In another antitumor research study, Jain et al. tested the intravenous toxicity profiles alongside antitumor efficacy of docetaxel (DTX) formulated in PHY/Plu‐F108 and PHY/Plu‐F108/PEG nanoparticles.^103^ In both the non‐PEG and PEGylated formulations, the loading of DTX converted the mesophase from *Pn3m* to H_2_ structure, presumably attributed to its lipophilic partition. Following an intravenous dose in a rat model, both PHY‐based hexosomes were shown to alleviate the vehicle‐ and drug‐associated toxicity markers (including injection site tail necrosis, nephrotoxicity, and hepatotoxicity) as typically observed with the commercial Taxotere formulation (DTX/polysorbate 80/ethanol) even when coadministered with prophylactic corticosteroids. This proof‐of‐concept study essentially highlights the viability of the PEGylated PHY nanoparticles in producing better systemic tolerance in parallel to more efficient tumor volume reduction in the breast cancer‐induced rat model: ≈81% (DTX‐PHY/Plu‐F108/PEG) versus 51% (DTX‐PHY/Plu‐F108) and 47% (Taxotere) over a 10 d monitoring. This goes in line with the concurrent in vitro tolerability profile showing that the blank PHY hexosomes were nontoxic at doses up to 10 µg mL^−1^ for 48 h.

Despite that both lamellar and nonlamellar lipid‐based systems were introduced simultaneously in the 1960s, liposome‐based nanomedicine has first led the pharmaceutical revolution and currently reaches successful commercialization of at least 15 liposomal products.^104^, ^105^, ^106^ Popular examples include the intramuscular inactivated hepatitis A virus, Epaxal (1993), intravenous doxorubicin, Doxil (1995), and epidural morphine sulfate, DepoDur (2004).^106^ More recently, the translational opportunities for nonlamellar LLC‐based technology are foreseen as Camurus (founded in 1991 and headquartered in Sweden) promisingly drives their proprietary FluidCrystal technologies toward human clinical trials and regulatory approval.^107^ The development pipeline features a range of LLC‐based depot or nanoparticle formulations currently being tested for multiple indications through human clinical trials (**Table** **3**). In particular, the FluidCrystal injection depot is developed to provide an extended therapeutic effect (up to months) via a single subcutaneous injection, where the lipids transform into the desired LC gel structures upon contact with fluids and degrade slowly in the tissue. On the other hand, the FluidCrystal nanoparticles are designed either for parenteral injection or as a topical spray to deliver lipophilic and/or biodegradation‐sensitive therapeutic molecules. Given that the genuine formulation, structural and biosafety profiles of these FluidCrystal products are kept confidential at this stage, it is challenging to outline the exact elements that have led to successful commercial proposition for these nonlamellar phase systems. The current understanding is that, these nanostructured nonlamellar LLC phases are developed to overcome the suboptimal performance of the commonly used micelle, emulsion, and liposome systems in terms of local and systemic tolerability, encapsulation efficiency, physical instability, manufacturing and excipient costs, and more importantly, sustained‐release control.^108^, ^109^ Interestingly, Otte et al. recently revealed the in vivo transformation of a H_2_ phase precursor depot formulation injected subcutaneously to a rat model, and showed that an earlier LC phase transformation (day 1 vs day 3) in conjunction with reduced matrix viscosity is associated with higher drug release and hence, increased bioavailability parameters.^110^ How the kinetics of LC formation in the body affects tissue tolerability has yet to be clarified. Nevertheless, successful advancement to Phase 3 trials as well as final approval and registration with the buprenorphine subcutaneous injection depot products (CAM2038) certainly attests the internal applicability of the nonlamellar LLC materials. The unique nanostructure‐enabled functionality of LLC systems is anticipated to form the next generation of nanotherapeutic entity for internal therapy.

**Table 3:**
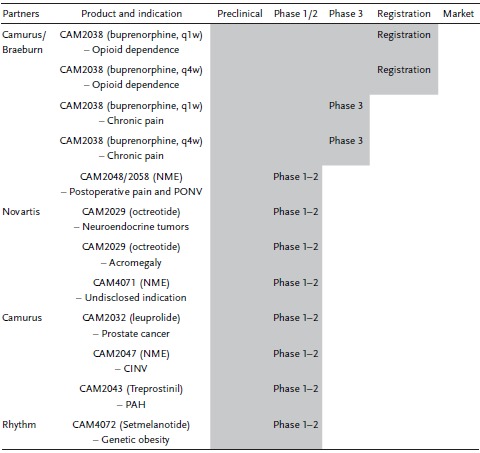
Pipeline of investigational products based on FluidCrystal lipid‐based liquid crystalline technologies of Camurus. Abbreviations: q1w (once a week), q4w (once every four weeks), NME (new molecular entity), CINV (chemotherapy‐induced nausea and vomiting), PONV (postoperative nausea and vomiting), and PAH (pulmonary arterial hypertension).^107^

## 5 Conclusions and Perspectives

The unique internal nanostructures of LLC self‐assemblies, such as the symmetrical bicontinuous inverse cubic (V_2_) and the anisotropic inverse hexagonal (H_2_) network, have presented unprecedented therapeutic values in diverse medical disciplines. State‐of‐the‐art technology, in particular synchrotron SAXS and neutron scattering facilities, as well as cryogenic transmission electron microscopy, has enabled high‐resolution morphological and structural characterization of these exotic nanostructures. To date, fabrication of LLC nanoparticles has gradually evolved from the high‐energy bulk processing techniques (including ultrasonication and homogenization) to the less energy‐demanding hydrotrope dilution and solvent evaporation methods, and more recently to the high‐precision microfluidic‐controlled drop‐by‐drop formation. In recent years, there is a clear impetus on the advancement of LLC technology progressing from material design toward biological and clinical translations. Fostered by the concerted efforts between material scientists and cellular biologists, research is in progress to delineate the systemic biointeractions of LLC materials from the perspectives of hematology and immunology, cellular association (which encompasses adhesion, uptake, and distribution), and tissue tolerability. Through a good collection of biointeraction studies of LLC nanoparticles using human and animal cell lines or blood specimens, this review has focused on what the authors see as the major research questions pertaining to their structure–biofunction relationships.

### 5.1 Does Contact with Blood Plasma Alter the Internal Architecture of LLC Domains?

To date, the existing phase behavior profiling using SAXS is mostly limited by the detection sensitivity, where lipid dispersions at relatively high dilutions (i.e., within the biocompatible concentration range of 10^1^–10^2^ µg mL^−1^) failed to provide sufficient contrast for distinguished scattering patterns to ascertain the underlying nanostructures. Nevertheless, feasible SAXS analyses of LLC phase behaviors in cell culture or blood media (at >10^4^ µg mL^−1^ lipid levels) provide compelling evidence that biological fluids can cause alteration to the lattice dimensions of LLC mesophases, and such lattice changes are dependent on the type of formulation additives. These changes likely underestimate the impact on structure in more dilute systems. Taking the example of an MO/CA and an MO/MCT system, contact with cell culture media and human plasma provoked noticeable increase in their lattice dimensions. In other cases based on the most commonly studied MO or PHY lipids, dispersion in either artificial or natural biological fluids generated mesophases that are different from that in water or in simple phosphate buffer. Based on these discrepancies, it seems necessary to establish phase diagrams of LLC systems using serum‐relevant media to more realistically interpret their phase behaviors in a biologically mimicking environment. This inherently has a direct impact on the interpretation of drug release and cellular uptake performance especially when geometrical control serves as the major mechanism fundamental to the biointeraction properties.

### 5.2 Does the Nanostructure of LLC Particles Determine Their Immunogenic Functions?

Despite the fact that cubic and hexagonal periodicities do exist in natural membranes for specific enzymatic and homeostatic functions, a number of in vitro studies have revealed that “traditional” cubosomes stabilized by Pluronic F127 are capable of eliciting the cell stress response via a single dose treatment. A prominent example is the PHY‐based cubosomes, which progressively elevated the proinflammatory markers in primary mouse splenocytes (i.e., IFN‐α and iNOS) without a clear resolution within 24 h, in addition to upregulating representative complement markers in human plasma extracts (including anaphylatoxin, C5a and membrane attack complex, SC5b‐9). However, not all LLC particles displaying similar nanostructure are alike in their immunogenicity. MO‐based cubosomes appeared to be mildly immunogenic where they transiently triggered the cell stress markers that gradually subsided within a few hours of exposure. It remains elusive as to whether the immunogenic effect is predominated by the digestibility nature of the different lipids or their fusogenic characteristics. However, replacing the nonionic Pluronic polymer with an anionic stabilizer (e.g., citrem) for surface coating was effective in mitigating the complement activation. This can possibly be due to the phase change from H_2_ to L_2_ phase structures (with increasing negative curvatures), or simply owing to other nonstructure‐related mechanisms rendered by the anionic surface agent such as improved biological camouflage. In order to clearly identify the immune safety and phagocytic susceptibility of these LLC nanoparticles, it certainly warrants more serological and (pre‐)clinical evidences to distinguish the role of lipid type, nanostructure, particle shape, and surface property.

### 5.3 Is There a Link between the Nanostructure of LLC Matrices and Their Cellular Interactions?

The nanostructure or phase behavior of LLC materials do play a partial but critical role in dictating the nanoparticle–cell interactions. For “traditional” LLC systems stabilized by the Pluronic linear copolymers (e.g., F127 and F108), a number of findings confirmed a general consensus that LLC nanoparticles with higher negative curvatures are less destructive toward cell membranes, presumably due to their lower degrees of lipid mixing. This is strongly corroborated by the observations that V_2_ mesophases induced relatively higher cytotoxic and hemolytic effects than the more negatively curved H_2_ and L_2_ phase analogues across different cell lines and red blood cells of different species. Such a toxicity trend can possibly correlate with the viscoelastic behaviors of the nanoparticles (where V_2_ is more “stiff” than H_2_) in tandem with the membrane disrupting capability of different lipid types (e.g., PHY is aggressive toward disrupting the cell nuclei membrane and blocking the plasma membrane turnover that is essential for macropinocytotic activities). However, such an interpretation is not absolute when an LLC formulation is complicated by the incorporation of phospholipid additives, change of stabilizers, and modification of the surface charge. The complex interplay among these multiple variables makes it a challenge to identify the predominating factor affecting cellular interactions. Some phospholipid dopants (e.g., CA, DLPC, and DSPC) were capable of enhancing the tolerability of some LC lipids, whereas others (e.g., DPPS) resulted in remarkably reduced cell viability. The choice of stabilizers other than the conventional Pluronic chains (such as anionic citrem and nonlinear brush copolymers) also changes the safety window of LLC nanoparticles. It is highlighted that a balance between the surface coverage and internal matrix insertion of the polymeric molecules has a significant control over the lipid molecular mobility and thus, the tendency of component transfer. With respect to charge‐dependent cytotoxicity, LLC nanoparticles with a low positive or low negative surface charge are generally better tolerated by tissue cells.

It is noteworthy that the existing cytotoxicity studies have mostly focused on dispersed LLC nanoparticles with average hydrodynamic diameters of ≤400 nm. Bearing in mind that some LLC systems may self‐assemble into larger structures in the micrometer scale, the effect of size on the interactions of LLC particles and mammalian cells represents a missing gap in the current LLC drug delivery research. Ironically, the current knowledge on cytotoxicity and biocompatibility profiles of LLC materials are built mostly from observations on cancerous cell lines subjected to a single‐dose treatment under static or nonflow experimental conditions. The interactions of LLC nanoparticles with healthy vascular and other tissue cells are almost neglected. This raises questions as to whether the existing findings are translatable for clinical significance, given that the physiological conditions are not adequately mimicked in terms of cell types, tissue dimensions, and blood flow variations. To make better sense of the LLC‐based therapeutic modality, future research directions need to be guided by more physiologically representable experimental models (such as flow‐integrated, 3D healthy vs diseased tissue‐mimicking models). The incomplete understandings of how different lipid‐based nanostructures interact with our bodily cells forecasts the need for a newly designed in vitro–in vivo investigation to bring LLC technology to its full therapeutic potential. Systematic in vivo investigations in terms of biotransformation, biodistribution, tissue clearance, and nanoparticle‐induced toxicity following different administration routes are undeniably important directions of future studies in the area of LLC nanomaterials.

## Conflict of Interest

The authors declare no conflict of interest.
